# The noncoding function of NELFA mRNA promotes the development of oesophageal squamous cell carcinoma by regulating the Rad17‐RFC2‐5 complex

**DOI:** 10.1002/1878-0261.12619

**Published:** 2020-01-28

**Authors:** Jiancheng Xu, Guangchao Wang, Wei Gong, Shichao Guo, Dan Li, Qimin Zhan

**Affiliations:** ^1^ State Key Laboratory of Molecular Oncology National Cancer Center/National Clinical Research Center for Cancer/Cancer Hospital Chinese Academy of Medical Sciences and Peking Union Medical College Beijing China; ^2^ Laboratory of Molecular Oncology Peking University Cancer Hospital and Institute Beijing China

**Keywords:** ESCC, NELFA mRNA, Rad17, RFC2‐5, USF2

## Abstract

Recently, RNAs interacting with proteins have been implicated in playing an important role in the occurrence and progression of oesophageal squamous cell carcinoma (ESCC). In this study, we found that NELFA mRNA interacts with Rad17 through a novel noncoding mode in the nucleus and that the aberrant expression of *USF2* contributed to the upregulation of *Rad17* and *NELFA*. Subsequent experiments demonstrated that the deletion of NELFA mRNA significantly decreased ESCC proliferation and colony formation *in vitro*. Moreover, NELFA mRNA knockdown inhibited DNA damage repair and promoted apoptosis. Mechanistic studies indicated that NELFA mRNA regulated the interaction between Rad17 and RFC2‐5, which had a major impact on the phosphorylation of CHK1, CHK2 and BRCA1. NELFA mRNA expression was consistently elevated in ESCC patients and closely related to decreased overall survival. Taken together, our results confirmed the critical role of the noncoding function of NELFA mRNA in ESCC tumorigenesis and indicated that NELFA mRNA can be regarded as a therapeutic target and an independent prognostic indicator in ESCC patients.

## Graphical abstract

NELFA mRNA interacts with Rad17 through a novel noncoding mode. NELFA mRNA knockdown significantly decreased oesophageal squamous cell carcinoma (ESCC) proliferation and colony formation. Moreover, NELFA mRNA knockdown inhibited DNA damage repair and promoted apoptosis. Mechanistic studies indicated that NELFA mRNA regulated the interaction between Rad17 and RFC2‐5. NELFA mRNA expression was consistently elevated in ESCC patients and closely related to decreased overall survival.

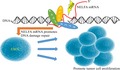

**Abbreviations**
ASO: antisense oligonucleotide
CoIP: co‐immunoprecipitation
DSB: DNA double‐strand break
EdU: 5‐ethynyl‐2′‐deoxyuridine
EMSA: electrophoretic mobility shift assay
ENCODE: encyclopedia of DNA elements
ESCC: oesophageal squamous cell carcinoma
FCM: flow cytometry
GAPDH: glyceraldehyde‐3‐phosphate dehydrogenase
GEPIA: gene expression profiling interactive analysis
IR: ionizing radiation
lncRNAs: long noncoding RNAs
qRT‐PCR: quantitative real‐time polymerase chain reaction
RACE: rapid amplification of cDNA ends
RIP: RNA immunoprecipitation
RPISeq: RNA–protein interaction prediction
SD: standard deviation
TCGA: The Cancer Genome Atlas

## 1 Introduction

Oesophageal cancer accounts for one of the leading causes of cancer‐related deaths worldwide (Alsop and Sharma, 2016). Approximately 70% of oesophageal cancer patients in the world are Chinese, of which over 90% have ESCC (Domper Arnal *et al.*, 2015; Huang and Yu, 2018). Despite recent technological advancements in early diagnosis and treatment, the 5‐year survival rate of patients is still poor (Masuike *et al.*, 2018; Yoshimizu *et al.*, 2018). At present, the specific mechanisms of oesophageal cancer onset and progression remain to be further defined. Above all, it is essential to investigate the molecular mechanisms involved in ESCC development and progression, thus facilitating new treatment strategies for ESCC patients.

Human *Rad17*, homologous to *Schizosaccharomyces pombe rad17*, plays an important role in DNA damage repair by forming a protein complex with RFC2‐5, which is vital for cell survival under genotoxic stress (Bao *et al.*, 1999; Bao *et al.*, 1998; Griffith *et al.*, 2002). Rad17 is essential for cell growth and chromosomal integrity and has weak ATPase activity required for binding to chromatin (Lindsey‐Boltz *et al.*, 2001). The Rad17‐RFC2‐5 complex participates in the ATR/ATM‐mediated phosphorylation of genes located downstream of the DNA damage response (Bao *et al.*, 2001; Medhurst *et al.*, 2008). In addition, the Rad17‐RFC2‐5 complex, as a loader complex, facilitates Rad9‐Hus1‐Rad1 loading onto chromatin (Hirai and Wang, 2002; Takeishi *et al.*, 2015). In this process, the phosphorylation of Rad17 at Ser 635 and Ser 645 is essential for the interaction between the Rad17‐RFC2‐5 complex and Rad9‐Hus1‐Rad1 (Bao *et al.*, 2001; Bermudez *et al.*, 2003; Medhurst *et al.*, 2008; Zou *et al.*, 2002). Previous studies have reported that human *Rad17* is overexpressed in diverse cancers, including colon carcinoma, breast cancer, pancreatic cancer, gastric cancer and non‐small‐cell lung cancer (Bao *et al.*, 1999; Fredebohm *et al.*, 2013; Sasaki *et al.*, 2001; Zhou *et al.*, 2013). The human protein NELFA, a component of the NELF complex, negatively regulates RNA polymerase II transcription elongation and is highly expressed in fetal tissues compared to adult tissues (Bastide *et al.*, 2016). Previous studies have shown that NELFA is involved in the transcription initiation and translation elongation of HIV (Natarajan *et al.*, 2013; Pagano *et al.*, 2014; Ping and Rana, 2001). Furthermore, NELFA is associated with the phenotype of Wolf–Hirschhorn syndrome (Kerzendorfer *et al.*, 2012). Until now, there has been no research on the correlation between *NELFA* and ESCC.

In our study, we found that a noncoding function of NELFA mRNA facilitated ESCC growth by interacting with Rad17. In addition, we confirmed that the expression of NELFA mRNA and Rad17 was higher in ESCC cells than in immortalized oesophageal epithelium cells, which was partially resulted from overexpression of the transcription factor USF2. Moreover, NELFA mRNA knockdown with unchanged NELFA protein expression levels achieved by specific antisense oligonucleotides (ASOs) significantly inhibited ESCC cell proliferation, colony formation and DNA damage repair and promoted ESCC cell apoptosis *in vitro*. Further mechanistic investigations indicated that NELFA mRNA exerted a noncoding function on DNA damage repair by regulating the interaction between Rad17 and RFC3/4. Most importantly, we demonstrated that the high expression level of NELFA mRNA was closely related to poor patient survival and might be viewed as an independent prognostic indicator for oesophageal cancer patients.

## 2 Materials and methods

### 2.1 Cell lines

The human ESCC cell lines KYSE30 and KYSE180 were obtained from Y. Shimada of Kyoto University. KYSE30 and KYSE180 cells were cultured in RPMI 1640 medium (Gibco, Thermo Fisher Scientific, Carlsbad, CA, USA) supplemented with 10% FBS. The immortalized oesophageal epithelium cell line NE3 was cultured in a 1 : 1 mixture of EpiLife and defined keratinocyte serum‐free medium (all from Gibco). All cell lines were grown at 37 °C in humidified air with 5% CO_2_. The source of all cell lines have been recently authenticated and tested for mycoplasma contamination, and no cross‐contaminated cell lines and mycoplasma contamination were found.

### 2.2 RNA immunoprecipitation assay

RNA immunoprecipitation (RIP) was carried out using an EZ Magna RIP Kit (Millipore, Bedford, MA, USA) according to the manufacturer's instructions. Briefly, KYSE30 and KYSE180 cells were lysed with RIP lysis buffer, and the supernatants were transferred to another tube for use. Fifty microlitres of protein A/G magnetic beads were incubated with anti‐Rad17 at room temperature for 30 min and then washed twice. The above supernatants were immunoprecipitated with beads/anti‐Rad17 complex in 900 µL of RIP Immunoprecipitation Buffer at 4 °C overnight. Finally, the immunoprecipitated RNA was detected by RT‐PCR.

### 2.3 RNA pull‐down

First, biotinylated RNA was transcribed by a MEGAscript™ T7 Transcription Kit (Invitrogen, Carlsbad, CA, USA) *in vitro*. Fifty picomoles of biotinylated RNA was heated at 95 °C for 2 min in an RNA structure buffer (10 mm Tris/HCI pH7.0, 10 mm MgCl_2_, 0.1 m KCl) and then cooled down at a rate of 1 °C per minute, during which the RNA secondary structure was restored. Subsequently, folded RNA was mixed with cell extracts in binding buffer and incubated for 30 min at room temperature. Fifty microlitres of blocked streptavidin agarose beads (GE, Marlborough, MA, USA) was added into each reaction and incubated for 15 min at room temperature. Finally, the beads were boiled in 2× SDS loading buffer, and the supernatants were detected by western blot analysis.

### 2.4 Rapid amplification of cDNA ends

A SMARTer^®^ rapid amplification of cDNA ends (RACE) 5′/3′ Kit was used to clone the 5′/3′ cDNA ends of NELFA (Clontech, Kusatsu, Shiga, Japan). A QIAquick^®^ Gel Extraction Kit was used to purify PCR‐amplified DNA fragments after agarose gel electrophoresis (Qiagen, Valencia, CA, USA). Purified DNA fragments were cloned into the pRACE vector (Clontech), and the 5′/3′ DNA of the NELFA sequence was identified by Sanger sequencing.

### 2.5 ChIP

ChIP was performed according to the manual of the ChIP Kit (CST, Boston, MA, USA). Briefly, KYSE30 and KYSE180 cells were cross‐linked with 1% formaldehyde for 10 min and terminated with the addition of 0.125 m glycine solution. DNA was fragmented into pieces ranging from 150 to 900 bp by ultrasonication. Then, 15 µg of DNA fragments was immunoprecipitated with an anti‐USF2 antibody or a normal rabbit IgG antibody. Immunoprecipitated DNA was purified and analysed by quantitative real‐time PCR (qRT‐PCR). The primers used for ChIP are listed in Table S1. Information on the antibodies is described in Table S2.

### 2.6 Electrophoretic mobility shift assay

Biotin‐labelled probes were synthesized by Invitrogen. Electrophoretic mobility shift assay (EMSA) was performed with a Gelshift™ (Active Motif, Carlsbad, CA, USA) Chemiluminescent EMSA Kit according to the manufacturer's suggestions. The probes used for EMSA are listed in Table S1.

### 2.7 Co‐immunoprecipitation

Equal numbers of cells were dissolved in Pierce™ IP lysis buffer (Thermo Scientific™) for 15 min on ice, followed by centrifugation for 15 min at 16 000 ***g***. Two micrograms of antibodies was added to each experiment and incubated overnight at 4 °C. Fifty microlitres of protein A/G sepharose beads was added to each experiment and incubated for 2 h at 4 °C. A subsequent immunoblot assay was used to authenticate the immunoprecipitates.

### 2.8 RNA *in situ* hybridization assay

RNA *in situ* hybridization (ISH) probes targeting NELFA mRNA were designed and synthesized by Advanced Cell Diagnostics (Newark, CA, USA). The expression of NELFA mRNA was detected by using an RNAscope^®^ 2.5 HD Assay—Brown (Advanced Cell Diagnostics) according to the manufacturer's instructions.

### 2.9 Small interfering RNA and ASO transfection

Aliquots of 1 × 106 cells were seeded into 60‐mm dishes and incubated in humidified air with 5% CO_2_ at 37 °C. Twenty‐four hours later, the small interfering RNA (siRNA; 50 µm), ASO (50 µm) and negative control (50 µm) were separately transfected into cells with Lipofectamine 2000 reagent (Invitrogen) according to the manufacturer's instructions. Knockdown efficiency was determined by qRT‐PCR or western blot analysis. Detailed sequence information is listed in Table S3.

### 2.10 qRT‐PCR

Total RNA was extracted using TRIzol reagent and then subjected to reverse transcription with TransScript First‐Strand cDNA Synthesis SuperMix (TransGen Biotech, Beijing, China). Quantitative PCR was performed utilizing TB Green^TM^ Premix Ex Taq^TM^ II (TaKaRa, Dalian, China), and relative gene expression was determined on an ABI PRISM 7900HT Sequence Detection System. The relative expression levels of the target genes were determined based on the 2^−∆∆Ct^ formula, and the human β‐actin transcript level was regarded as an internal control. Information on the primers is described in Table S1.

### 2.11 Western blot analysis

Cells were lysed in 1× RIPA buffer containing protease inhibitor. The cell lysate was separated by SDS/PAGE and transferred to polyvinylidene difluoride (PVDF) membranes. After blocking the PVDF membranes with Tris‐buffered saline containing Tween‐20 (TBST) containing 5% milk for 1 h at room temperature, the membranes were incubated with primary antibodies at 4 °C overnight. Then, the membranes were washed with TBST three times before incubation with secondary antibodies for 1 h at room temperature. Finally, membrane chemiluminescence was detected on an ImageQuant LAS 4000 (GE). Information on the antibodies is described in Table S2.

### 2.12 Colony formation assay

A total of 10^3^ KYSE30 and KYSE180 cells transfected with the ASO were seeded into a six‐well plate. After 12 days, colonies were stained with 0.5% crystal violet and imaged.

### 2.13 Cell proliferation assay

A total of 2 × 10^3^ KYSE30 and KYSE180 cells transfected with the ASO were seeded into a 96‐well E‐Plate, and the xCELLigence Real‐Time Cell Analyzer (RTCA)‐MP System (ACEA, Agilent Technologies, Inc., Santa Clara, CA, USA) was used to monitor cell proliferation.

### 2.14 Ethynyl deoxy uridine incorporation assay

A total of 2 × 10^4^ KYSE30 and KYSE180 cells were seeded into 96‐well plates 24 h prior to transfection with the ASO. After transfection for 48 h, 5‐ethynyl‐2′‐deoxyuridine (EdU) labelling was performed according to the manufacturer's instructions provided in the Cell‐Light EdU Apollo567 In Vitro Kit (RiboBio, Guangzhou, China).

### 2.15 γH2AX immunofluorescent staining

A total of 2 × 10^4^ KYSE30 and KYSE180 cells were seeded into 96‐well plates 24 h prior to transfection with the ASO. After transfection for 48 h, KYSE30 and KYSE180 cells were treated with 15 Gy ionizing radiation (IR). After 3 h, γH2AX immunofluorescent staining was performed according to the manufacturer's instructions provided in the OxiSelect™ DNA Double‐Strand Break (DSB) Staining Kit (CELL BIOLABS, INC, San Diego, CA, USA).

### 2.16 Cell apoptosis assay

A total of 3 × 10^5^ KYSE30 and KYSE180 cells were seeded into six‐well plates 24 h prior to transfection with the ASO. After transfection for 48 h, KYSE30 and KYSE180 cells were treated with 15 Gy IR. After 3 h, KYSE30 and KYSE180 cells were stained with propidium iodide and annexin V (BD Biosciences, San Jose, CA, USA). Flow cytometry (FCM) was used to determine the percentage of apoptotic cells.

### 2.17 Statistical analysis

spss 18.0 (SPSS, IBM, Armonk, NY, USA) and graphpad prism 5 (GraphPad Software Inc, LaJolla, CA, USA) software were used for statistical analyses. Student's *t*‐test was used to analyse differences in gene expression and the correlation between NELFA mRNA expression and clinicopathological characteristics. Univariate and multivariate Cox proportional hazards regression methods were used to analyse potential prognostic factors. The Kaplan–Meier method and the log‐rank test were applied to estimate overall survival. Each *P* value was two‐sided, and *P* < 0.05 was considered statistically significant. All experiments were performed in at least three independent replicates, and the data are presented as the mean ± standard deviation (SD).

## 3 Results

### 3.1 Rad17 interacts with NELFA mRNA in ESCC cells

**Figure 1:**
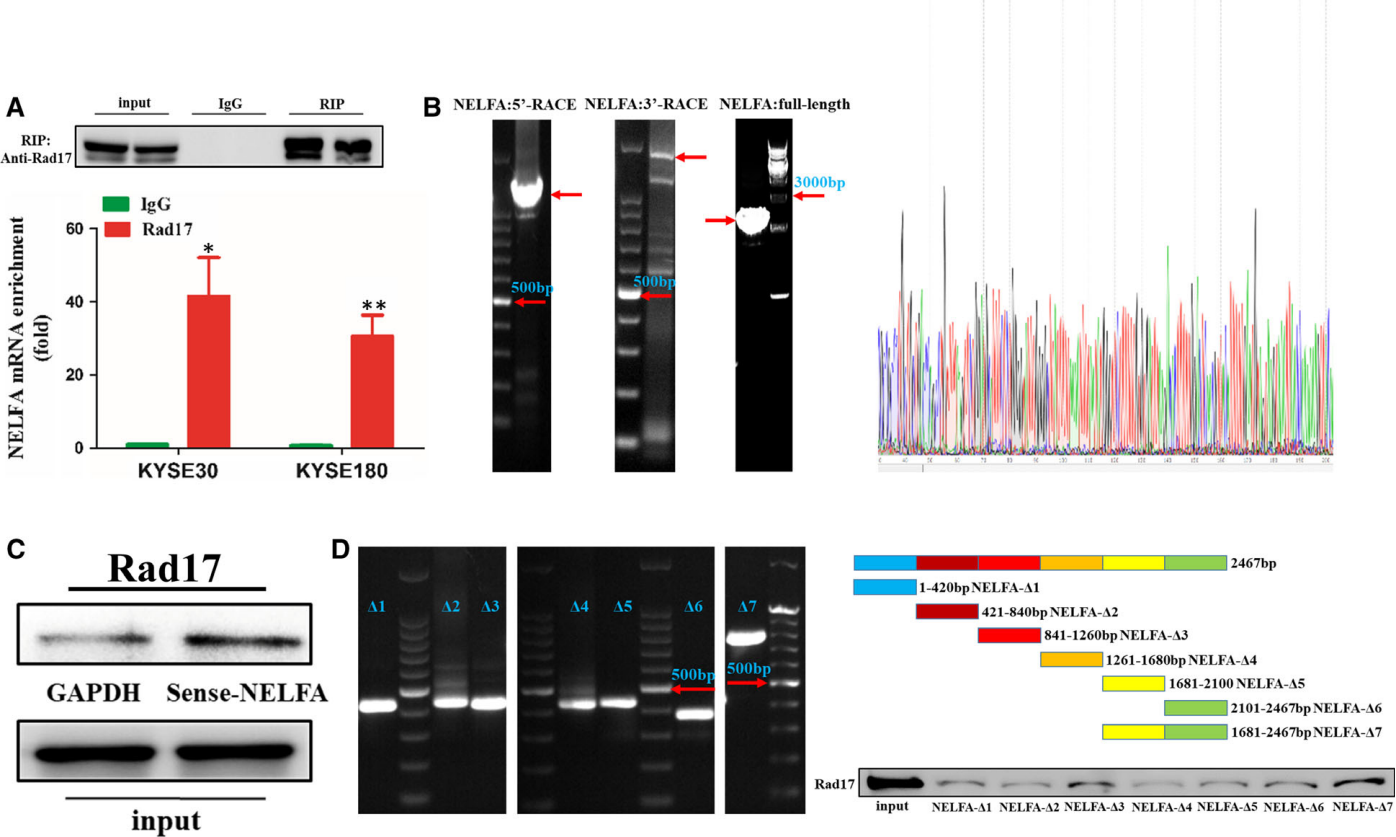
Rad17 interacts with NELFA mRNA in ESCC cells. (A) Rad17 antibody enriched NELFA mRNA in KYSE30 and KYSE180 cells. (B) NELFA mRNA sequence was verified by RACE assays. The red arrow indicates the target fragment. (C) RNA pull‐down assays were used to detect the interaction between Rad17 and NELFA mRNA in KYSE30 cells. (D) Different NELFA mRNA truncations were used to pull down Rad17. *P < 0.05, **P < 0.01. Significance was determined by Student's *t*‐test. Error bars indicate SD. All experiments were performed for three times.

Little is known about the potential interaction between DNA damage checkpoints and long noncoding RNAs (lncRNAs). In our research, Rad17‐enriched RNAs in ESCC cells were extracted by RIP with anti‐Rad17 and then analysed by high‐throughput sequencing. We discovered 355 lncRNAs and 399 mRNAs that combined with Rad17. Then, RIP‐qPCR was used to identify these deep sequencing data to ascertain which RNAs could directly bind to Rad17. As indicated in Fig. 1A, NELFA mRNA was highly enriched by the Rad17 antibody in ESCC cells.

In addition, the 5′ and 3′ end sequences of NELFA mRNA were cloned by RACE, and the overlapping regions of these two sequences were identical (Fig. 1B). Subsequent sequence alignment indicated that the cloned sequences were consistent with NCBI reference sequences NM_005663.4. As shown in Fig. 1C, the RNA pull‐down assay proved that Rad17 could directly combine with NELFA mRNA. Additionally, the RNA–protein interaction prediction instrument RPISeq showed that Rad17 and NELFA mRNA likely interact with each other, with an RF or SVW score > 0.5 (Muppirala *et al.*, 2011). Furthermore, we constructed different truncations of NELFA mRNA based on RPISeq prediction. The specific truncation of NELFA mRNA that mainly interacted with Rad17 was a 1681–2467 bp truncation (containing the 3′ nontranslated region and the partial ORF region), as determined by RNA pull‐down assays (Fig. 1D). In summary, the above results suggest that NELFA mRNA interacts with Rad17 in ESCC cells.

### 3.2 Transcription factor USF2 regulates the expression of Rad17 and NELFA

**Figure 2:**
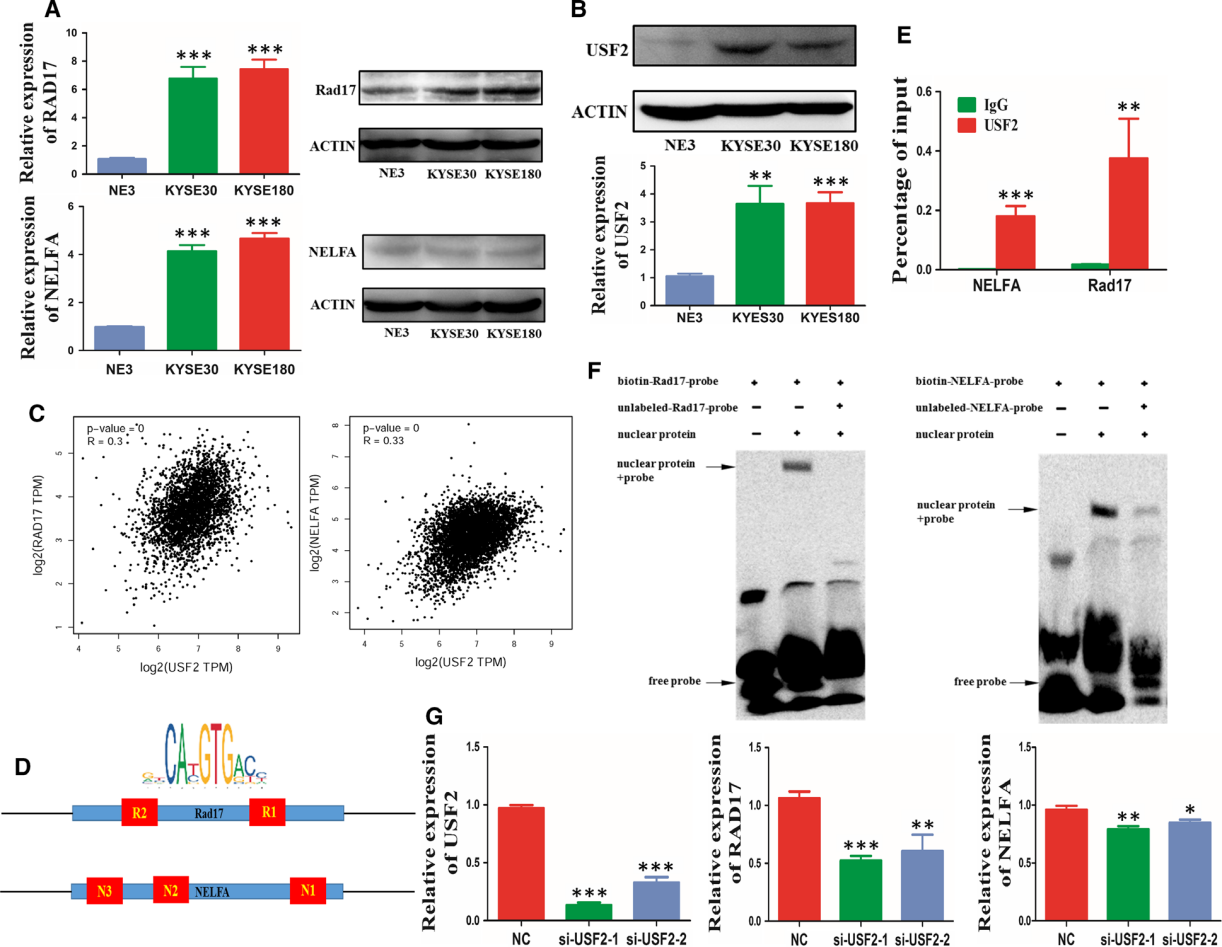
USF2 regulates the expression of Rad17 and NELFA. (A) qRT‐PCR and western blot assays were used to detect Rad17 and NELFA expression in ESCC cell lines (KYSE30 and KYSE180) and compared with that in the normal oesophagus cell NE3. (B) Mutations in USF2 in the TCGA and USF2 expression were determined by qRT‐PCR and western blot analyses. (C) GEPIA was used to analyse the correlation between USF2, Rad17 and NELFA expression. (D) Analysis of the USF2‐binding motif in the Rad17 and NELFA promoter regions. (E) ChIP‐qPCR assays were carried out to detect USF2 expression at the Rad17 and NELFA promoter regions. (F) EMSA was used to identify the binding motif region. The nuclear protein (containing USF2) was extracted from nucleus and incubated with R2 and N1 EMSA probes, respectively. (G) Rad17 and NELFA expression was detected by qRT‐PCR in KYSE30 cells transfected with USF2 siRNAs. *P < 0.05, **P < 0.01 and ***P < 0.001. Significance was determined by Student's *t*‐test. Error bars indicate SD. All experiments were performed for three times.

As depicted in Fig. 2A, the mRNA expression levels of Rad17 and NELFA were significantly higher in ESCC cells than in NE3 cells. In addition, the protein level of Rad17 was obviously higher in ESCC cells than in NE3 cells. Interestingly, the protein level of NELFA in ESCC cells was comparable to that in NE3 cells.

To identify the potential regulators of *Rad17* and *NELFA* overexpression in ESCC, we utilized the Encyclopedia of DNA Elements (ENCODE) database to select possible transcription factors that could bind to the promoters of *Rad17* and *NELFA* (Davis *et al.*, 2018). We found that USF2 might be the upstream regulator of *Rad17* and *NELFA* (Fig. S1A). The Cancer Genome Atlas (TCGA) data suggested that *USF2* exhibited copy number amplification. RT‐qPCR and western blot results also indicated that USF2 was upregulated in ESCC cells (Fig. S1B and Fig. 2B). Furthermore, through gene expression profiling interactive analysis (GEPIA), we found that the gene expression of USF2 was positively related to RAD17 or NELFA in multiple tumours (Fig. 2C) (Tang *et al.*, 2017). Next, we analysed the *Rad17* and *NELFA* promoter regions and discovered that Rad17 contains two USF2‐binding motifs (R1 and R2), while NELFA contains three (N1, N2 and N3) upstream of its transcriptional start sites (Fig. 2D). ChIP‐qPCR assays further demonstrated that the R2 site of the Rad17 promoter and the N1 site of the NELFA promoter served as the binding site of USF2 (Fig. 2E).

The above results were further confirmed by an EMSA, as shown in Fig. 2F. We then knocked down USF2 in KYSE30 and KYSE180 cells, and Rad17 and NELFA expression was decreased (Fig. 2G). In conclusion, these results demonstrate that USF2 serves as an upstream regulator of Rad17 and NELFA overexpression in ESCC cells.

### 3.3 NELFA mRNA affects the proliferation of ESCC cells

**Figure 3:**
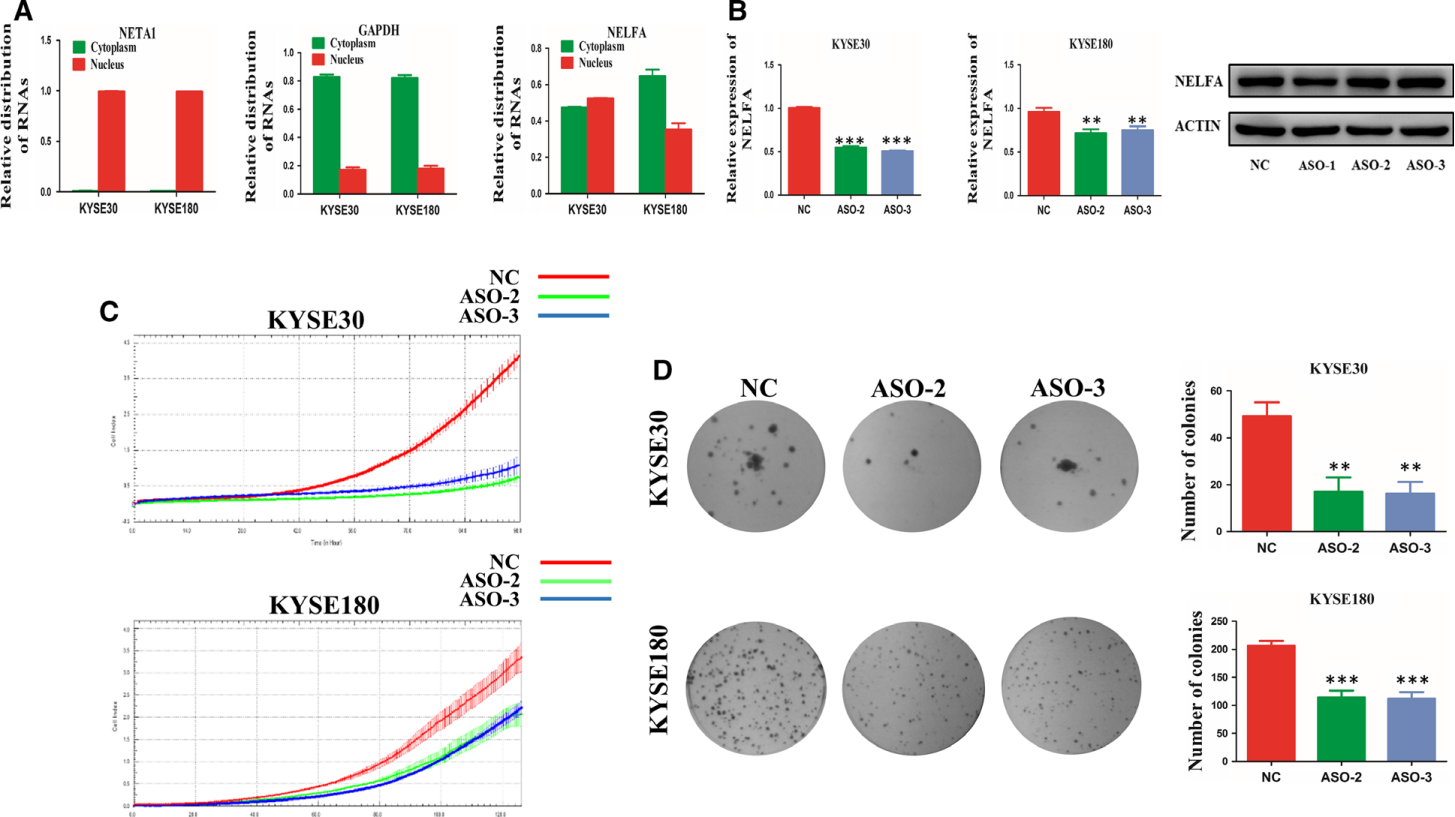
NELFA mRNA impacts the growth of ESCC cells. (A) Distribution of NELFA mRNA in ESCC cell lines. (B) NELFA mRNA expression was detected by qRT‐PCR and western blot analyses in KYSE30 and KYSE180 cell lines transfected with the NELFA mRNA ASO. (C) RTCA assays were used to examine KYSE30 and KYSE180 cell proliferation after transfection with the NELFA mRNA ASO. (D) Decrease in the clone formation ability of KYSE30 and KYSE180 cell lines transfected with the NELFA mRNA ASO. **P < 0.01 and ***P < 0.001. Significance was determined by Student's *t*‐test. Error bars indicate SD. All experiments were performed for three times.

Rad17 is mainly located in the cell nucleus and plays an important role in DNA damage repair. Therefore, the distribution of NELFA mRNA in cells was investigated. RNAs extracted from the nuclear and cytoplasmic fractions were subjected to RT‐qPCR to identify NELFA mRNA distribution independently. NELFA mRNA was expressed in the cytoplasm and in the nucleus (Fig. 3A). Then, we used two different ASOs, ASO‐2 and ASO‐3, to specifically downregulate NELFA mRNA expression in the cell nucleus with no impact on the expression level of NELFA protein (Fig. 3B). Thereafter, we performed an RTCA assay. NELFA mRNA knockdown in the cell nucleus inhibited the proliferation of KYSE30 and KYSE180 cells (Fig. 3C). Likewise, clone formation assays indicated that the clone formation ability of KYSE30 and KYSE180 cells decreased dramatically when nuclear NELFA mRNA was downregulated (Fig. 3D). Taken together, these results show that NELFA mRNA regulates ESCC cell growth.

### 3.4 NELFA mRNA affects DNA damage repair and the apoptosis of ESCC cells

**Figure 4:**
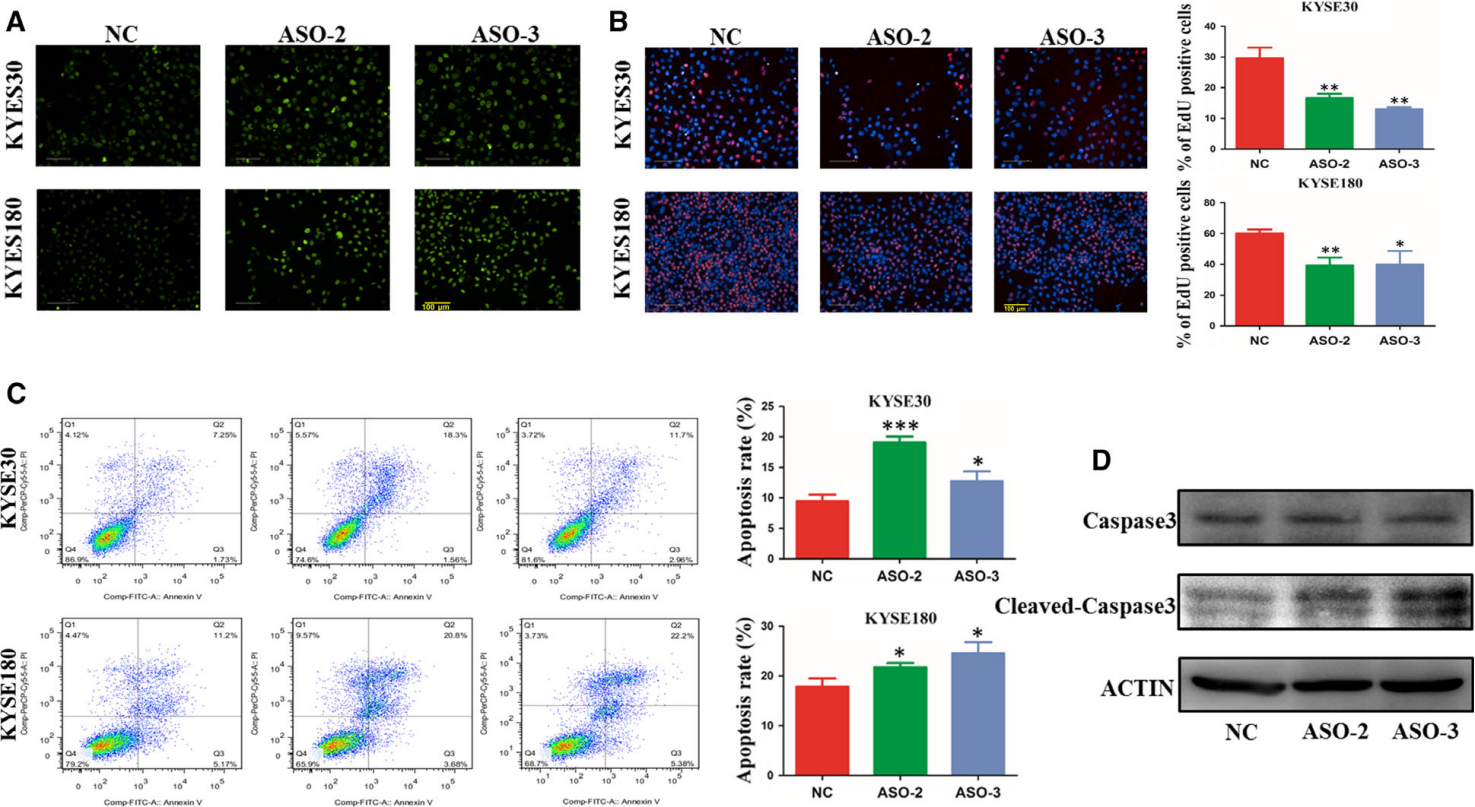
NELFA mRNA affects DNA damage repair and the apoptosis of ESCC cells. (A) DNA damage repair was indicated by immunostaining with γH2A.X (IR 15 Gy). (B) EdU dye assays were used to monitor DNA damage repair (IR 15 Gy). (C) Flow cytometric analysis of the effect of NELFA mRNA knockdown on cell apoptosis. (D) Western blot analysis was used to detect the apoptosis‐related proteins caspase‐3 and cleaved caspase‐3 after NELFA mRNA knockdown. Scale bar = 100 μm. *P < 0.05, **P < 0.01 and ***P < 0.001. Significance was determined by Student's *t*‐test. Error bars indicate SD. All experiments were performed for three times.

Previous studies have shown that the major function of Rad17 is in DNA damage repair. However, whether NELFA mRNA interacts with Rad17 and participates in DNA damage repair remains unknown. To elucidate the impact of NELFA mRNA on DNA damage repair, we constructed a DNA damage repair model induced with IR. Then, we detected γH2AX immunofluorescence in KYSE30 and KYSE180 cells with or without NELFA mRNA knockdown after IR treatment. As indicated in Fig. 4A, dramatically increased γH2AX immunofluorescence was observed following NELFA mRNA knockdown in the nucleus, which implied that NELFA mRNA knockdown could restrain DNA DSB repair. Similarly, EdU assays demonstrated that NELFA mRNA knockdown significantly inhibited DSB repair (Fig. 4B). Herein, we have to point out that the DNA synthesis of a small proportion of cells subjected to IR might not be blocked, their EdU signal might be inevitably included in the results of our EdU assays.

Given that DNA damage repair inhibition might lead to increased cell apoptosis, we next explored the impact of NELFA mRNA knockdown on ESCC cell apoptosis by FCM. As expected, a significantly increased proportion of apoptotic cells was observed in the NELFA mRNA knockdown group after IR (Fig. 4C). Furthermore, the protein level of cleaved caspase‐3 was significantly increased after NELFA mRNA knockdown (Fig. 4D). Overall, these results suggest that NELFA mRNA facilitates ESCC cell growth by regulating DNA damage repair and apoptosis.

### 3.5 NELFA mRNA is required for activation of the DNA damage response

**Figure 5:**
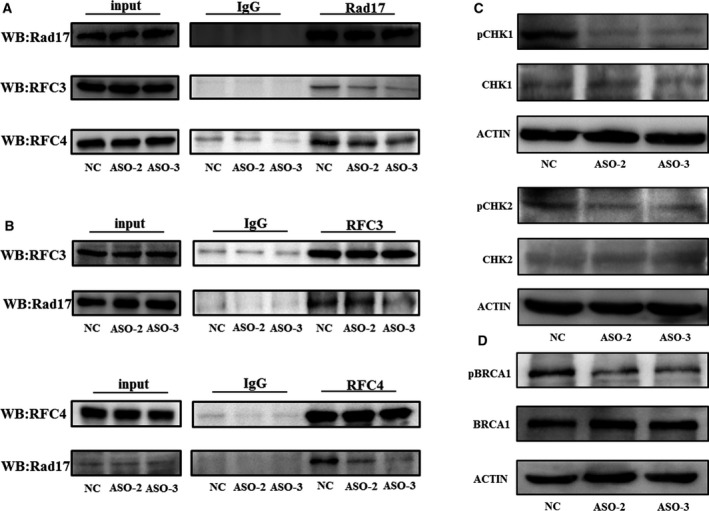
NELFA mRNA is required for activation of the DNA damage response. (A, B) A CoIP assay was performed to monitor the interaction between Rad17 and RFC3/4 after transfection with the NELFA mRNA ASO. (C) Western blot analysis of the changes in CHK1 and CHK2 phosphorylation after transfection with the NELFA mRNA ASO. (D) Western blot analysis of the change in BRCA1 phosphorylation after NELFA mRNA knockdown followed by IR treatment (15 Gy).

Some studies have established that a proportion of noncoding RNAs participate in the formation of protein complexes. Furthermore, previous studies have revealed that Rad17 interacts with RFC2‐5, particularly RFC3/4. Based on these facts, we performed Rad17 co‐immunoprecipitation (CoIP) assays to identify whether NELFA mRNA plays an important role in the formation of the Rad17‐RFC2‐5 complex through RFC3/4. Western blot assays revealed that the interaction between Rad17 and RFC3/4 was inhibited following NELFA mRNA knockdown (Fig. 5A). RFC3/4 CoIP further verified that NELFA mRNA is essential for the formation of the Rad17‐RFC2‐5 complex (Fig. 5B).

Genomic stability is persistently threatened by genotoxic stress from either internal or environmental sources in eukaryotic cells. To protect the integrity of genomes, cells have developed a series of DNA damage response programmes. Previous findings have indicated that Rad17‐RFC2‐5 primarily participates in the DNA damage response induced by genotoxic stress and triggers the phosphorylation of ATM/ATR substrates, especially BRCA1, CHK1 and CHK2. Western blot assays revealed that the deletion of NELFA mRNA significantly decreased the phosphorylation of CHK1 and CHK2, which are critical for initiation of the DNA damage repair pathway (Fig. 5C). Furthermore, NELFA mRNA knockdown followed by IR inhibited the phosphorylation of BRCA1, which is mainly responsible for the homologous recombination of DSBs (Fig. 5D). Taken together, the above results suggest that NELFA mRNA is extensively involved in the DNA damage response pathway by facilitating the formation of the Rad17‐RFC2‐5 complex.

### 3.6 NELFA mRNA could be regarded as a prognostic indicator for ESCC

To explore the clinical significance of NELFA mRNA in ESCC, we obtained tissue microarrays including 93 ESCC and 67 para‐carcinoma tissues from Outdo Biotech Co. Then, RNAscope ISH assays were applied to detect NELFA mRNA expression through the tissue microarrays.

**Figure 6:**
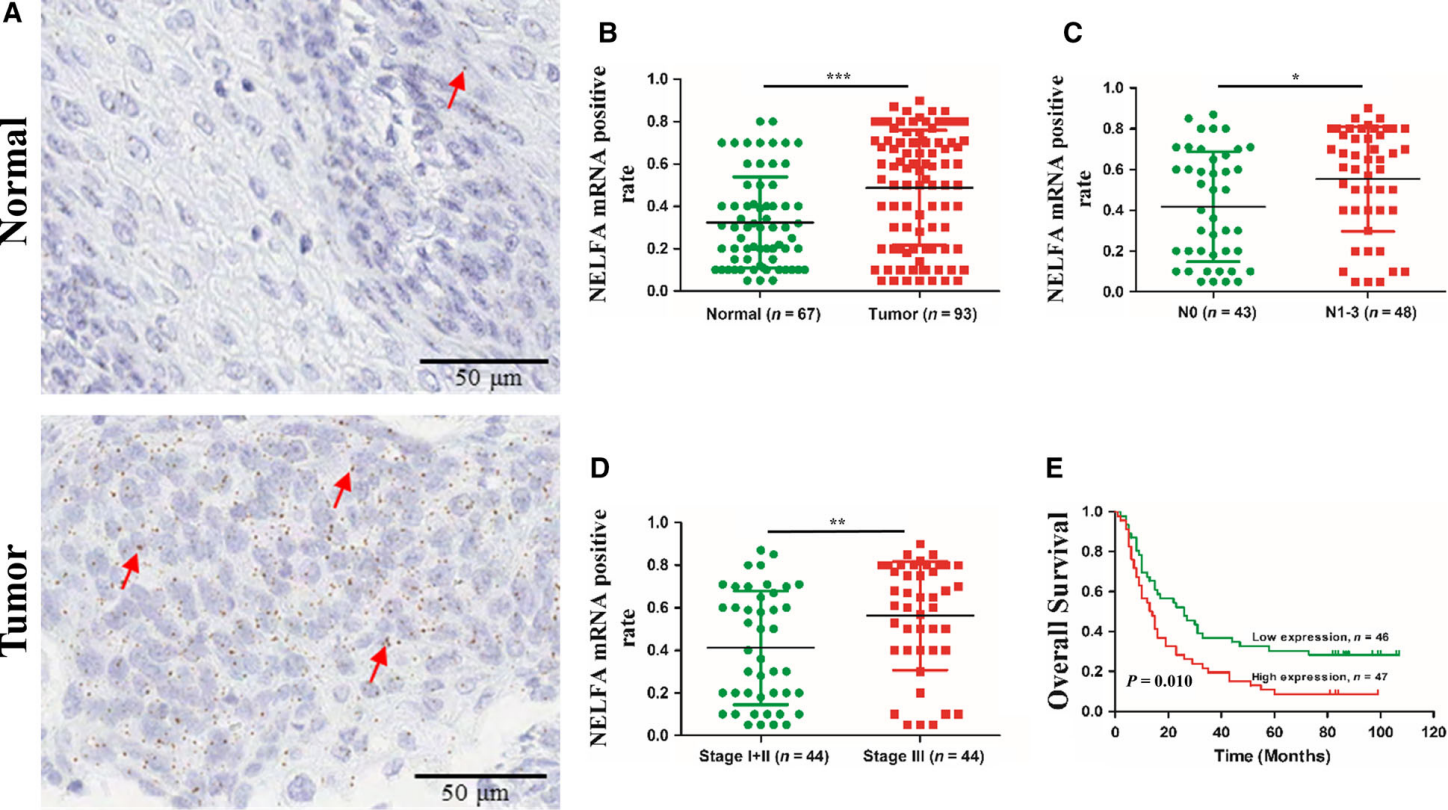
NELFA mRNA can be used as a biomarker for ESCC prognosis. (A, B) RNAscope ISH assays were used to detect NELFA mRNA expression in the tissue microarray. The red arrow indicates the NELFA mRNA signalling point. Normal *n* = 67, tumour *n* = 93. (C) The correlation between the NELFA mRNA‐positive rate and lymph node metastasis. N0 *n* = 43, N1–3 *n* = 48. (D) The correlation between the NELFA mRNA‐positive rate and TNM stage. Stage I + II *n* = 44, stage III *n* = 44. (E) Kaplan–Meier survival analysis of ESCC patients' overall survival based on the NELFA mRNA‐positive rate in the tissue microarray. Low expression *n* = 46, high expression *n* = 47. Scale bar = 50μm.*P < 0.05, **P < 0.01 and ***P < 0.001. Significance was determined by Student's *t*‐test.

As shown in Fig. 6A,B, NELFA mRNA expression was significantly upregulated in ESCC tissues compared with para‐carcinoma tissues. Next, we used Student's *t*‐test to further analyse the correlation between NELFA mRNA expression and the clinicopathological characteristics of ESCC. As shown in Table 1, the NELFA mRNA‐positive rate was correlated with tumour lymph node metastasis (Fig. 6C) and tumour–node–metastasis (TNM) stage (Fig. 6D). Furthermore, we also investigated the relationship between the NELFA mRNA‐positive rate and ESCC prognosis. Overall survival analysis using the log‐rank test indicated that a higher NELFA mRNA‐positive rate was associated with reduced overall survival in ESCC patients (Fig. 6E).

**Table 1:** The correlation between NELFA mRNA expression and clinicopathological characteristics in tissue microarray of 93 ESCC patients.

	Total cases	*P* value^a^
Gender		
Male	68	0.254
Female	25	
Age		
< 66	46	0.657
≥ 66	47	
T^b^		
T1–2	15	0.262
T3–4	75	
N^c^		
N0	43	**0.017**
N1–2	48	
Stage^d^		
Stage I + II	44	**0.008**
Stage III	44	
Overall survival		
Dead	76	**0.010**
Alive	17	

a Statistical significant results (in bold).

b T stage, three missing values.

c Lymph node metastasis, two missing values.

d Five missing values.

Moreover, to further ascertain the prognostic role of NELFA mRNA expression in ESCC patients, univariate and multivariate Cox regression analyses were conducted. As shown in Table 2, the univariate Cox regression analysis confirmed that the NELFA mRNA‐positive rate (*P* = 0.010), gender (*P* < 0.001), lymph node metastasis (*P* < 0.001), T stage (*P* = 0.009) and TNM stage (*P* < 0.001) were vital prognostic factors. The multivariate analysis further revealed that NELFA mRNA expression was an independent prognostic biomarker (*P* = 0.047). Taken together, these results suggest that NELFA mRNA overexpression might be a vital factor in ESCC progression and prognosis.

**Table 2:** Univariate and multivariate Cox regression analyses of clinic pathologic factors associated with overall survival in 93 ESCC patients. CI, confidential interval; HR, hazard ratio.

Variable	Univariate analysis		Multivariate analysis	
	HR (95% CI)	*P* value^a^	HR (95% CI)	*P* value^a^
NELFA mRNA‐positive rate	1.83 (1.159–2.898)	**0.010**	1.602 (1.007–2.550)	**0.047**
Age (< 66 years vs. ≥ 66 years)	0.909 (0.579–1.429)	0.680		
Gender (male vs. female)	2.084 (1.180–3.682)	**0.011**		
N (N = 0 vs. N = 1–3)	2.325 (1.452–3.724)	**< 0.001**		
T (T = 1–2 vs. T = 3–4)	2.061 (1.193–3.558)	**0.009**		
Stage (I + II vs. III)	2.602 (1.678–4.035)	**< 0.001**	2.408 (1.556–3.727)	**< 0.001**

a Statistical significant results (in bold).

## 4 Discussion

Previous studies have shown that lncRNAs play crucial roles in the occurrence and development of ESCC (Wu *et al.*, 2015; Yao *et al.*, 2016; Zhang *et al.*, 2017). In our study, the most notable finding was that the noncoding function of NELFA mRNA plays an important role in oesophageal cancer progression. We used RIP‐Seq assays to identify noncoding RNAs that directly bind to Rad17. Then, RIP‐qPCR and RNA pull‐down assays were used to verify the interaction between Rad17 and RNAs. Finally, we found that NELFA mRNA interacts with Rad17. Gilot *et al*. reported that the noncoding function of TYRP1 mRNA facilitated melanoma development (Gilot *et al.*, 2017). Combining the results of previous and current studies, we speculated that the noncoding functions of mRNAs might play an important role in various types of cancer.

Previous studies have reported that Rad17 expression is elevated in colon carcinoma, breast cancer, pancreatic cancer, gastric cancer and non‐small‐cell lung cancer and is correlated with tumour cell onset and progression (Bao *et al.*, 1999; Fredebohm *et al.*, 2013; Wang *et al.*, 2001). NELFA is an essential element of the NELF complex that negatively regulates the elongation of RNA polymerase II‐mediated transcription (Gilchrist *et al.*, 2008; Jadlowsky *et al.*, 2014; Stadelmayer *et al.*, 2014). Previous studies have indicated that NELFA is related to HIV infection and Wolf–Hirschhorn syndrome (Garber and Jones, 1999; Kerzendorfer *et al.*, 2012; Zhang *et al.*, 2007). Until now, very few studies have explored the relationship between NELFA and cancer. In our research, we identified that the expression of NELFA mRNA and Rad17 was higher in ESCC cells than in normal epithelial cells. Moreover, we also revealed that the upregulated transcription factor USF2, at least partly, led to the upregulation of NELFA mRNA and Rad17. USF2, a member of the basic helix–loop–helix leucine zipper transcription factor family, activates transcription through pyrimidine‐rich initiator elements and E‐box motifs (5‐CACGTG‐3) and participates in the regulation of multiple cellular processes (Belew *et al.*, 2018; Ghosh *et al.*, 1997; Takahashi *et al.*, 2001). Previous studies have shown that USF2 is closely related to ESCC (Xiang *et al.*, 2012). However, the molecular mechanism by which USF2 is involved in ESCC is still unclear. In our study, USF2 exhibited copy number amplification in a variety of cancers according to the TCGA database. RT‐qPCR and western blot results also indicated that *USF2* was upregulated in ESCC cells. ChIP‐qPCR and EMSA demonstrated that USF2 could bind to the promoter regions of *Rad17* and *NELFA*. Moreover, USF2 knockdown led to the reduced expression of *Rad17* and *NELFA*. Taken together, these results indicate that the molecular mechanism by which USF2 is involved in ESCC onset and progression might partially be explained by the increased expression of *Rad17* and *NELFA*.

Our results provide novel insight into the important role of *NELFA* in the onset and progression of ESCC through a noncoding function. Given that previous studies have suggested that Rad17 is mainly located in and functions in the cell nucleus (Post *et al.*, 2003), we first investigated the distribution of NELFA mRNA in cells. By separating the nuclear and cytoplasmic fractions, we found that NELFA mRNA was expressed in both the cytoplasm and nucleus. Subsequently, to explore the function of NELFA mRNA, we designed two different ASOs to specifically silence NELFA mRNA in the cell nucleus with no impact on the protein expression level.

Antisense oligonucleotides are short oligonucleotides that localize to the nucleus and provide a pathway for gene silencing by the RNase H pathway. As we all know, RNase H, specifically located in the nucleus, could degrade all the RNAs in DNA and RNA double heterozygous strands. Furthermore, in ASO knockdown group, the NELFA mRNA expression level decreased by a certain percentage, which was comparably equivalent with the proportion of NELFA mRNA distributed in the nucleus. In view of this, we silenced NELFA mRNA expression in the nucleus with no impact on the protein expression level in our experiment. Besides, ASO2 and ASO3 were designed to respectively combine with the exon 8 and exon 11 of NELFA mRNA. NELFA mRNA knockdown inhibited ESCC cell proliferation and clone formation ability. In addition, silencing NELFA mRNA in the cell nucleus also suppressed DNA damage repair and facilitated ESCC cell apoptosis. This result implies that NELFA mRNA regulates ESCC cell growth, at least partially, through its regulation of DNA damage‐associated apoptosis. Considering that NELFA mRNA overexpression or knockout would inevitably influence the expression of NELFA protein, we did not perform these assays in ESCC cells.

Many studies have suggested that noncoding RNAs participate in the formation of protein complexes (McHugh *et al.*, 2015; Munschauer *et al.*, 2018; Wen *et al.*, 2018). Previous results have suggested that Rad17 interacts with RFC2‐5 (Majka and Burgers, 2004). The interaction between Rad17 and RFC2‐5 is involved in ATM‐ and/or ATR‐mediated substrate phosphorylation through a complicated regulatory network (Foray *et al.*, 2003; Shiotani *et al.*, 2013; Zou *et al.*, 2002). Our data indicated that NELFA mRNA knockdown inhibited the interaction between Rad17 and RFC3/4. We also found that NELFA mRNA knockdown inhibited the phosphorylation of CHK1, CHK2 and BRCA1, which would have an impact on the DNA damage repair pathway. In conclusion, NELFA mRNA promotes ESCC initiation and progression by facilitating the formation of the Rad17‐RFC2‐5 complex. However, the underlying mechanism of the ATM‐ and/or ATR‐mediated DNA damage repair signalling pathway regulated by Rad17 and NELFA mRNA needs further exploration.

Oesophageal cancer is one of the deadliest cancers, with a 5‐year survival rate of ~ 20% (Li *et al.*, 2018). In our study, we used RNAscope ISH assays to detect NELFA mRNA expression in an ESCC tissue microarray. NELFA mRNA was significantly upregulated in ESCC tissues compared with para‐carcinoma tissues (*P* < 0.001). In addition, the NELFA mRNA expression level was positively correlated with tumour lymph node metastasis (*P* < 0.05) and TNM stage (*P* < 0.01). The overall survival analysis indicated that high NELFA mRNA expression was associated with reduced overall survival in ESCC patients. Moreover, univariate and multivariate Cox regression analyses further revealed that NELFA mRNA expression was an independent prognostic biomarker (*P* = 0.047). These results suggest that NELFA mRNA expression can be regarded as a biomarker for ESCC prognosis.

## 5 Conclusions

In summary, our results demonstrated that NELFA mRNA could interact with Rad17 in a noncoding mode and promote ESCC cell growth, at least partially, by facilitating DNA damage repair. In addition, upregulated NELFA mRNA was related to cancer progression and a poor prognosis. These results suggest a strategy for targeting NELFA mRNA as a therapeutic target for ESCC in the future.

## Conflict of interest

The authors declare no conflict of interest.

## Author contributions

QZ contributed to conceptualization, administered project, supervised the study and acquired funding. JX contributed to methodology; wrote, reviewed and edited the manuscript; curated the data; and developed the software. GW performed formal analysis and curated the data. WG contributed to visualization and curated the data. SG validated the study. DL participated in investigation.

## Ethics approval and consent to participate

Our research was approved by the Ethics Committee of Cancer Hospital Chinese Academy of Medical Sciences and Peking Union Medical College.

## Supporting information


**Fig. S1.** Transcription factor analysis of *Rad17* and *NELFA* in database.

**Table S1.** The list of primers.

**Table S2.** Information of antibodies.

**Table S3.** siRNAs, ASOs and EMSA probe sequence.

## Data Availability

Cistrome DB (http://cistrome.org/db/#/) ENCODE (https://www.encodeproject.org/) GEPIA (http://gepia.cancer-pku.cn/) RPISeq (http://pridb.gdcb.iastate.edu/RPISeq/) TCGA (https://www.cancer.gov/)
