# Supplementary figures and images for: The noncoding function of NELFA mRNA promotes the development of oesophageal squamous cell carcinoma by regulating the Rad17‐RFC2‐5 complex
Source: Mol Oncol. 2020 Jan 28;14(3):611–24. doi: 10.1002/1878-0261.12619 (PMC7053240; doi:10.1002/1878-0261.12619)

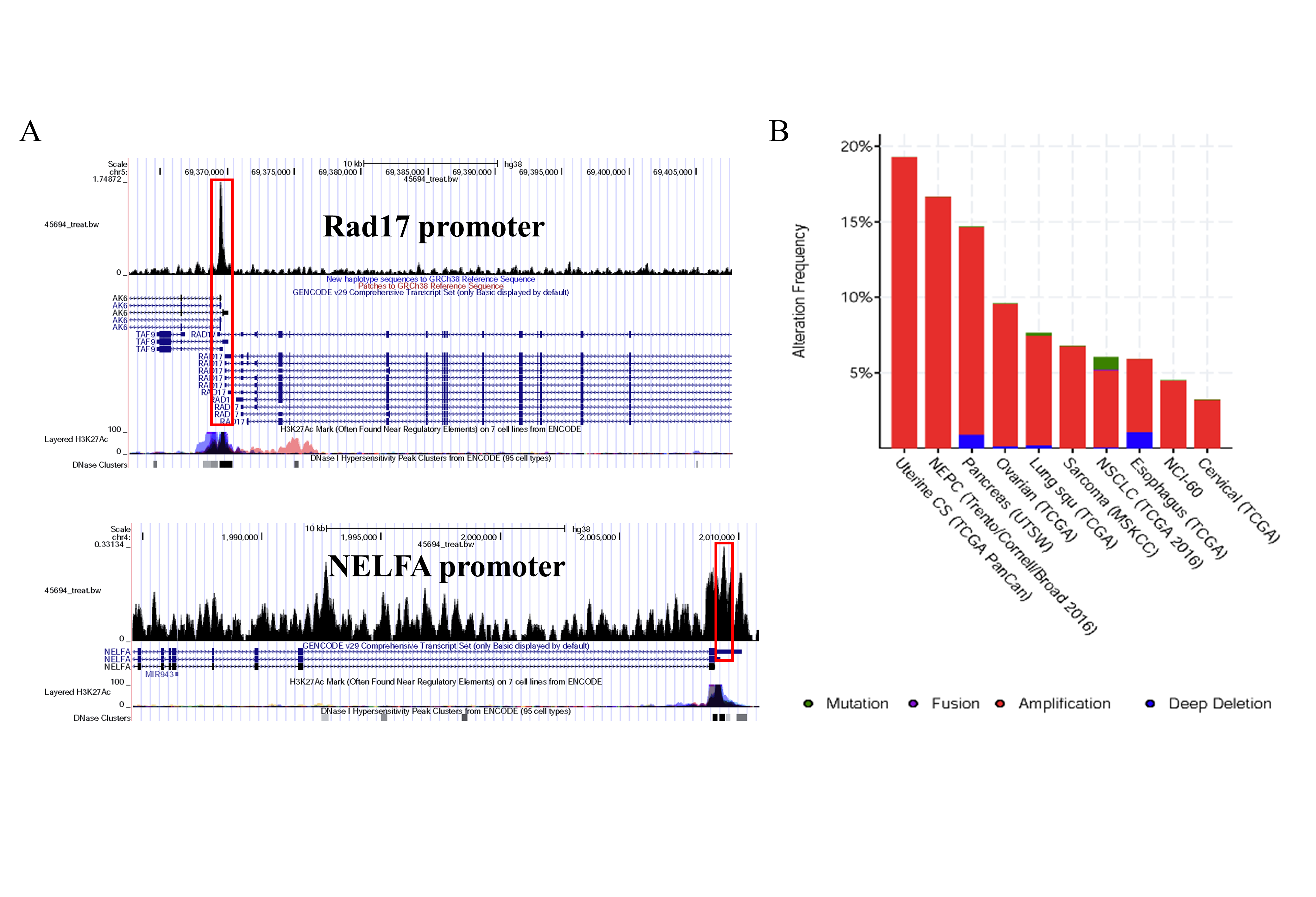

Supplement: Supplementary file 1 — Fig. S1. Transcription factor analysis of Rad17 and NELFA in database. [file MOL2-14-611-s001.tif]
