# Supplementary material for: The noncoding function of NELFA mRNA promotes the development of oesophageal squamous cell carcinoma by regulating the Rad17‐RFC2‐5 complex
Source: Mol Oncol. 2020 Jan 28;14(3):611–24. doi: 10.1002/1878-0261.12619 (PMC7053240; doi:10.1002/1878-0261.12619)
Supplement: Supplementary file 2 — Table S1. The list of primers. [file MOL2-14-611-s002.docx]

**Table S1** The list of primers

| **qPCR primers** | | |
| --- | --- | --- |
|  | **Forward Primer** | **Reverse Primer** |
| Rad17 | CCTTGCTCTACTAACCATTC | TTTCAATCTTCCAAAGTGTC |
| USF2 | TTGATGGAACCAGAACACCC | AGCTGGACGATCCAGTTGTT |
| NELFA | ACCCCCATCCCGCCTTCCAG | CTTCTCCGCTTCGCCTCTCG |
| ACTB | TGGCACCCAGCACAATGAAG | AAGCATTTGCGGTGGACGAT |
| **primers for ChIP** | | |
|  | **Forward Primer** | **Reverse Primer** |
| R1 | CCACCCACCTGGCCTATTTG | GATCCTCCCGCCTTCTCCTC |
| R2 | GGTGAGCAGGATGTTCGGAA | CCTGTAGAGCAAAGGGCACG |
| NE1 | CGGTATTTGGGTTTCTCCTA | CACACCACTGAACTCAGGCG |
| NE2 | GATTCTCCTGCCTCAGCCTCCT | GCCATCATAGTGAAACCCCGTT |
| NE3 | GGGTCACGAGTGCCCCTGGA | GCCCTGCGGAGCGAGCCTGC |
| **primers for RACE** | | |
|  | **3’RACE** | |
| NELFA-GSP | GATTACGCCAAGCTTCTGCTGCGGAAGGAACGAGGTGTGAA | |
|  | **5’RACE** | |
| NELFA-GSP | GATTACGCCAAGCTTTCAGGGACCCAAGTTTCTGCGTGGAC | |
