# Supplementary material for: The noncoding function of NELFA mRNA promotes the development of oesophageal squamous cell carcinoma by regulating the Rad17‐RFC2‐5 complex
Source: Mol Oncol. 2020 Jan 28;14(3):611–24. doi: 10.1002/1878-0261.12619 (PMC7053240; doi:10.1002/1878-0261.12619)
Supplement: Supplementary file 3 — Table S2. Information of antibodies. [file MOL2-14-611-s003.docx]

**Table S2** Information of antibodies

|  |  | **Western blot** | **ChIP** | **RIP** | **Co-IP** |
| --- | --- | --- | --- | --- | --- |
| Anti-Rad17 | abcam#ab50526 | 1:1000 |  | 2μg | 2μg |
| Anti-USF2 | abcam#ab125184 | 1:1000 | 2μg |  |  |
| Anti-Chk1 | abcam#ab40866 | 1:1000 |  |  |  |
| Anti-BRCA1 | abcam#213929 | 1:1000 |  |  |  |
| Phospho-Chk1 (Ser345) (133D3) Rabbit mAb | cell signaling technology#2348 | 1:1000 |  |  |  |
| Chk2 (D9C6) XP® Rabbit mAb | cell signaling technology#6334 | 1:1000 |  |  |  |
| Phospho-Chk2 (Thr68) (C13C1) Rabbit mAb | cell signaling technology#2197 | 1:1000 |  |  |  |
| Phospho-BRCA1 (Ser1524) Antibody | cell signaling technology#9009 | 1:1000 |  |  |  |
| Caspase-3 Antibody | cell signaling technology#9662 | 1:1000 |  |  |  |
| Cleaved Caspase-3 (Asp175) (5A1E) Rabbit mAb | cell signaling technology#9664 | 1:1000 |  |  |  |
| Anti-RFC3 | proteintech#11814-1-AP | 1:500 |  |  | 2μg |
| Anti-RFC4 | proteintech#10806-1-AP | 1:500 |  |  | 2μg |
| Anti-NELFA | proteintech#10456-1-AP | 1:500 |  |  |  |
| Monoclonal Anti-Actin antibody | Sigma-Aldrich #A4700 | 1:2000 |  |  |  |
