# Supplementary material for: The noncoding function of NELFA mRNA promotes the development of oesophageal squamous cell carcinoma by regulating the Rad17‐RFC2‐5 complex
Source: Mol Oncol. 2020 Jan 28;14(3):611–24. doi: 10.1002/1878-0261.12619 (PMC7053240; doi:10.1002/1878-0261.12619)
Supplement: Supplementary file 4 — Table S3. siRNAs, ASOs and EMSA probe sequence. [file MOL2-14-611-s004.docx]

**Table S3** siRNAs, ASOs and EMSA probe sequence

| **siRNAs** | |
| --- | --- |
|  | **target sequence** |
| si-USF2#1 | 5'-GGATCGTCCAGCTTTCGAA-3' |
| si-USH2#2 | 5'-GAGGCACGATTTGCCTATT-3' |
| **ASO** | |
|  | **target sequence** |
| NELFA#2 | 5'-AAACTTGGGTCCCTGAACAA-3' |
| NELFA#3 | 5'-AGAAGTACAAGCCCATGACC-3' |
| **EMSA probe** | |
|  | **Forward** |
| Bio-R2 | Bio-5'-GCGCCTCGCTCACGTGCCCTTTGCTCTACA-3'-Bio |
| R2 | 5'-GCGCCTCGCTCACGTGCCCTTTGCTCTACA-3' |
| Bio-NE1 | Bio-5'-TGTGGCCCACGCCTGAGTTCAGTGGTGTGA-3'-Bio |
| NE1 | TGTGGCCCACGCCTGAGTTCAGTGGTGTGA |
|  | **Reverse** |
| Bio-R2 | Bio-5'-TGTAGAGCAAAGGGCACGTGAGCGAGGCGC-3'-Bio |
| R2 | TGTAGAGCAAAGGGCACGTGAGCGAGGCGC |
| Bio-NE1 | Bio-5'- TCACACCACTGAACTCAGGCGTGGGCCACA -3'-Bio |
| NE1 | TCACACCACTGAACTCAGGCGTGGGCCACA |
